# Comparative profiling of neurological and biomarker status in type 1 diabetes and multiple sclerosis: A cross-sectional observational study

**DOI:** 10.1016/j.bbih.2026.101210

**Published:** 2026-03-03

**Authors:** Fátima Cano-Cano, Álvaro J. Cruz-Gómez, Lucía Forero, Almudena Lara-Barea, Elena Lozano-Soto, Raúl Espinosa-Rosso, Raúl Rashid-López, Eduardo Alcalde-Vílchez, Pablo Álvarez-Ramos, Manuel Aguilar-Diosdado, Ana I. Arroba, Javier J. González-Rosa

**Affiliations:** a Psychophysiology and Neuroimaging Group, Institute of Research and Biomedical Innovation of Cadiz (INiBICA), Spain; b Diabetes Mellitus Laboratory, Institute of Research and Biomedical Innovation of Cadiz (INiBICA)., Cádiz, Spain; c Department of Psychology, University of Cadiz, Spain; d Neurology Department, Puerta del Mar University Hospital, Cadiz, Spain; e Endocrinology and Metabolism Department, Puerta del Mar University Hospital, Cádiz, Spain; f Department of Ophthalmology, Puerto Real University Hospital, Cádiz, Spain

**Keywords:** Type 1 diabetes mellitus, Multiple sclerosis, MRI, OCT, Cognition, Neurodegeneration biomarkers

## Abstract

**Background and objectives:**

Type 1 diabetes mellitus (T1DM) and multiple sclerosis (MS) are considered chronic organ-specific autoimmune diseases. Their high prevalence, progressive nature, impact on daily life, the need for lifelong management, and increased co-occurrence have attracted research interest. However, the comparative evaluation of neurological and cognitive status in these diseases and their impact on multimodal neurodegenerative biomarkers remains underexplored.

**Methods:**

This cross-sectional observational study included 76 participants, including 18 patients with newly diagnosed T1DM, 26 patients with relapsing-remitting MS, and 20 healthy controls (HCs). Additionally, 12 patients with long-duration T1DM were included in exploratory analyses to investigate the effects of prolonged disease exposure. Comprehensive assessments included clinical and neuropsychological evaluations, cellular immune profiles testing, alongside advanced neuroimaging techniques, such as whole-brain and regional grey matter (GM) volumetry and optical coherence tomography, for retinal nerve fibre layer (RNFL) thickness. Serum neurofilament light chain (sNfL) and glial fibrillary acidic protein (GFAP) levels were measured.

**Results:**

Patients with newly diagnosed MS and T1DM exhibited cognitive and mood disturbances, elevated sNfL levels, and distinct immunological and biochemical profiles compared with HCs. Patients with MS showed significant thalamic volume reduction and RNFL thinning, whereas those with T1DM did not exhibit significant GM volume loss or RNFL thinning. However, patients with advanced T1DM presented with increased sNfL concentrations and a trend towards retinal thinning. Partial correlations, controlling for age, schooling, or intracranial volume, revealed that impaired cognitive processing speed was significantly associated with higher sNfL levels, increased retinal thinning, and a smaller left thalamus volume.

**Discussion:**

Our findings emphasize the shared and distinct mechanisms of neurodegeneration underlying cognitive dysfunction in patients with newly diagnosed MS and T1DM, suggesting that both diseases involve overlapping mechanisms of immune-mediated damage and neurodegenerative processes that can contribute to the future development of neurological disability.

## Highlights

• Blood marker sNfL were elevated in both T1DM and MS, indicating axonal damage.
• Retinal thinning occurs in early MS and late T1DM, but not in early diabetes cases.
• Grey matter loss is observed in newly diagnosed MS but not T1DM patients.
• Low cognitive speed relates to elevated NfL, thalamic atrophy, and retinal thinning.
• T1DM and MS may share neuro-immune damage underlying similar neurological complications.

## 1 Introduction

Multiple sclerosis (MS) and type 1 diabetes mellitus (T1DM) are chronic, autoimmune diseases affecting different organs, the central nervous system (CNS) myelin and pancreatic β-cells, respectively. Despite these differences, both diseases share immune-mediated damage mechanisms, systemic and neuroinflammatory processes, and progressive neurological complications beyond their classical definitions (Haki et al., 2024). Moreover, having one autoimmune disease increases the risk of developing other diseases, as is the case with T1DM and MS (Pozzilli et al., 2022; Tettey et al., 2015). These parallels provide a compelling rationale for comparative research into their neurological and cognitive effects, particularly in the early stages of disease. Despite demographics and clinical differences, their autoimmune origin has led researchers to identify environmental, genetic, and immunological similarities (Pozzilli et al., 2022; Tettey et al., 2015). A comprehensive comparison of clinical outcomes and neurological status between both chronic conditions is needed. Furthermore, converging research employing different brain magnetic resonance imaging (MRI) modalities has demonstrated that grey matter (GM) atrophy is an early and robust indicator of cognitive decline and that widespread changes in brain functional networks may modulate the manifestation of this cognitive dysfunction (Sumowski et al., 2018; Wood, 2021).

The critical impact of MS disease on the CNS is widely recognized, leading to demyelination, inflammatory, and neurodegenerative changes that generally involve multiple neurological signs or brain and spinal cord lesions that are separated in time, resulting in progressive disability (Filippi and Rocca, 2010; Haki et al., 2024). Moreover, cognitive impairment can emerge even in early stages of MS, affecting up to 70% of patients depending on the disease progression and significantly impacting quality of life (Morrow et al., 2023).

In contrast, the consequences of T1DM on the CNS remain poorly understood. Chronic CNS abnormalities, including changes in brain structure and vascular integrity, retinopathy and cerebral disturbances, have been systematically documented in T1DM patients (Umegaki, 2012; Wrighten et al., 2009). Various mechanisms, mainly insulin resistance and impaired insulin signaling, neuroinflammation, oxidative stress, autonomic and neurovascular dysfunction, and white matter (WM) and GM lesions, may further lead to cerebrovascular accidents and cognitive dysfunction in T1DM patients (Liu et al., 2020; Musen et al., 2018). Despite typically receiving little attention, there is growing evidence that T1DM may contribute to the early onset and accelerated progression of cognitive impairment (Jin et al., 2022; Shalimova et al., 2019).

Ocular involvement is a hallmark of both T1DM and MS, albeit with differing underlying mechanisms. In MS, optic neuritis and subsequent retinal axonal damage lead to measurable thinning of the retinal nerve fiber layer (RNFL), reflecting neuroaxonal injury (Sumowski et al., 2018). In T1DM, chronic dysglycemia and microvascular dysfunction drive retinal changes, including diabetic retinopathy, which may also contribute to RNFL thinning and neurodegeneration over time (Katsarou et al., 2017). Spectral-domain optical coherence tomography (SD-OCT) provides noninvasive, high-resolution imaging to detect these alterations, serving as a critical biomarker of neurodegeneration and assisting early detection (Cunha et al., 2022). When combined with blood-based biomarkers, such as neurofilament light chain (NfL), a marker of neuronal injury and neurodegeneration, and glial fibrillary acidic protein (GFAP), a marker of astrocyte dysfunction, this multimodal approach enhances the understanding of CNS pathology and highlights its potential diagnostic significance (Garzone et al., 2022). While NfL and GFAP are well-established diagnostic and prognostic markers in MS (Barro et al., 2022; Khalil et al., 2024), the results in diabetes are still scarce and inconclusive (Hernández et al., 2023; Pang et al., 2017). To date, studies examining brain structure integrity and neural biomarkers of neurodegeneration among patients with T1DM have yielded inconsistent findings.

Thus, for a comprehensive understanding of the similarities between T1DM and MS and their impact on the brain, an integrate multimodal approach is required. Here, we aimed to investigate blood profiles and neurodegeneration biomarkers, as well as retinal and brain GM volume integrity signatures to explore the factors associated with clinical and cognitive alterations. This integrative approach can not only advance our understanding of the underlying systemic correlates of cognitive dysfunction in both diseases but also aid in the early detection of patients at risk of clinical and cognitive impairments in the first stages of both autoimmune diseases.

## 2 Methods

### 2.1 Study participants

A total of 76 participants without any compromised data were retained in this cross-sectional observational study. All outpatients were recruited from the Puerta del Mar Universitary Hospital (HUPM) of Cádiz. In the case of diabetes, 18 patients diagnosed with T1DM following the American Diabetes Association (ADA) criteria (Care and Suppl, 2021) were initially enrolled, whereas in the case of MS, 26 patients diagnosed with clinically definite relapsing-remitting multiple sclerosis (henceforth MS) in accordance with the McDonald criteria (Polman et al., 2011) were selected.

The inclusion criteria for this study of T1DM patients were as follows: i) aged between 18 and 60 years; ii) diagnosed with T1DM within the past 4 years; iii) positive results for glutamic acid decarboxylase and tyrosine phosphatase autoantibodies; and iv) continuous insulin treatment. The inclusion criteria for MS patients were as follows: i) aged between 18 and 60 years; ii) diagnosed with MS within the past 4 years; iii) experienced mild physical disability (with an Expanded Disability Status Scale score equal to or less than 3.5 (Kurtzke, 1983); and iv) were free from relapses and steroid treatment for at least 2 months prior to the study.

Among the recruited patients with MS, 46.2% were receiving conventional moderate-efficacy disease-modifying therapies (DMT, interferon-beta or glatiramer acetate), whilst 53.8% were under high-efficacy DMTs (fingolimod, natalizumab, alemtuzumab, or ocrelizumab (Oreja-Guevara et al., 2024)). In the case of T1DM patients, treatment consisted in subcutaneous insulin therapy administered via multiple daily injection regimen, with a mean total daily insulin dose of 0.745 IU/kg/day.

Additionally, we included 20 participants with no history of neurological, psychiatric or immunological issues as the healthy control (HCs) group. The participants’ main characteristics are summarized in Table 1.

**Table 1:** Demographic, cellular and biochemical parameters.

Variables	HC (n = 20)	T1DM (n = 18)	MS (n = 26)	p-value
**Demographic parameters**				
**Age**	35,4 (6,71)	37,00 (12,66)	40,73 (10,34)	0,184
**Sex**				
**Male/female**	10/10	10/8	9/17	
**Female %**	50	44,45	65,38	-
**Disease duration (years)**	-	3,21 (1,5)	4,31 (2,91)	0,174
**Clinical parameters**				
**EDSS**	-	-	1,48 (1,36)	-
**HbA1c %**	-	6,9 (0,009)	-	-
**Cellular populations**				
**Leukocytes**	5,8 (1,19)	6,28(1,48)	6,15(2,76)	0,876
**Erythrocytes**	4,53 (0,31)	4,84 (0,45)	4,64 (0,44)	0,094
**Eosinophils %**	3,06 (2,61)	4,6(3,17)	2,88(1,98)	0,33
**Basophil %**	0,56 (0,27)	0,79 (0,27)	0,41 (0,31)	0,002^c^
**Monocytes %**	7,9 (1,52)	7,67 (1,83)	9,55 (2,93)	0,1
**Neutrophils %**	52,45 (7,89)	53,68(11,67)	60,93(15)	0,09
**Lymphocytes %**	35,78 (7,24)	33,22(9,89)	26,23 (13,43)	0,024
**Platelets**	245,82 (33,48)	223,73(37,16)	235,25 (66,94)	0,51
**Biochemical parameters**				
**Glucose (mg/dL)**	90,91 (12,98)	178,94(57,37)	89,79(15,09)	0,00^a,c^
**Cholesterol**	190,55 (33,88)	178,06(47,81)	174,73(30,95)	0,458
**Triglycerides**	71,4 (18,15)	80,53(39,84)	94,03(46,91)	0,43
**Hemoglobin**	13,35 (1,13)	10,18 (7,17)	13,9 (1,31)	0,008^c^
**Uric Acid**	5,61 (2,96)	3,96(0,73)	4,97 (4,10)	0,045
**Urea**	29,56 (6,5)	28,15(10,74)	31,3(9)	0,83
**Creatinine**	0,72 (0,13)	1,12(0,79)	0,75(0,18)	0,02^c^
**Serological measures (SIMOA)**				
**NfL (pg/ml)**	5.21 (2.18)	7.34 (3.28)	7.59 (4.06)	0.091^a,b^
**GFAP (pg/ml)**	68.90 (28.72)	68.46 (35.52)	60.71 (26.82)	0.533

*Abbreviations*: NfL = Neurofilament light chain; GFAP = Glial Fibrillary Acid Protein; SIMOA = Single Molecular Assay. Values are expressed as the mean and SD. ANOVA or Kruskal-Wallis test were performed to assess significant differences between groups (p values are included), and Bonferroni or Dunns post-hoc analysis were used for pair comparisons. a HCs vs T1DM (p < 0.05), b HCs vs MS (p < 0.05), c T1DM vs MS (p < 0.05).

All clinical and neuropsychological assessments, blood withdrawals, and MRI and OCT scans were conducted across up to three separate sessions, corresponding to different evaluations performed within the same individuals, with an average inter-session interval of 1.89 ± 1.72 months.

To evaluate the progression of clinical and serum biomarkers and for exploratory purposes, the study was ultimately amended to include an extra group of 12 T1DM patients who fulfilled the same inclusion criteria but had a longer disease duration, over 4 years from onset (T1DM advanced).

### 2.2 Standard protocol approvals, registrations, and patient consents

The research protocol for this study received approval from the Andalusian Biomedical Research Ethics Committee for MS participants (Reference: LFD-VIT-2018-01) and from the Biomedical Research Ethics Committee of Cádiz for T1DM individuals (Reference: 04-abr-PI22-01718). Before participation, all individuals provided informed written consent, adhering to the principles outlined in the Declaration of Helsinki.

### 2.3 sNfL and GFAP level analysis

The samples from all participants were collected in clot-activating separator tubes and allowed to clot at room temperature for 30 min. After this time, the tubes were centrifuged for 10 min at 1500×*g*, resulting in the extraction of the serum from the supernatant. The serum was then aliquoted and properly stored at −80 °C until analysis. All the measurements were conducted using highly sensitive single-molecule array (Simoa™) technology with the SR-X instrument by Quanterix Corporation (MA, United States). Serum levels of GFAP and NfL were assessed using the neurology 2-plex B assay (Quanterix, MA, United States). Data analysis was performed in accordance with the manufacturer's instructions and recommendations for serum biomarkers.

### 2.4 SD-OCT analysis

Retinal imaging was performed through SD-OCT by using a DRI OCT Triton (Topcon Medical Systems, Inc., Oakland, NJ, USA). SD-OCT images were obtained by qualifying operators from the Ophthalmology Department at HUPM in Cádiz via a 6 × 6 mm acquisition protocol centered in the fovea of both eyes. The precise position of the placement around the center of the optic disc was thoroughly examined. The scans were analyzed by using a 3-D segmentation algorithm to determine the RNFL thickness. Segmentation measures were acquired from the same region on each scan, and the average thicknesses of the different segments of the RNFL were carefully analyzed and compared between the study groups.

The presence of hyperreflective spots (HRS) was also evaluated. As previously reported (Vujosevic et al., 2017; Zhu et al., 2022), HRS are characterized by their punctate appearance and distinct boundaries within the retina. A key defining feature was their reflectivity, which was required to be at least equivalent to, or greater than, the signal intensity of the retinal pigment epithelium, thus differentiating them from background noise.

### 2.5 MRI data acquisition and neuropsychological evaluations

Brain MRI was performed with a 1.5 T scanner (Philips Medical System, Ingenia CX, Best, Netherlands). Two sagittal whole-brain sequences were acquired: a fluid-attenuated inversion recovery (FLAIR) 3D sequence (TR = 6000 ms; TE = 354 ms; flip angle = 180°, matrix = 196 × 256 × 160, and voxel size = 1.05 × 1.05 × 1 mm) and a T1-weighted 3D magnetization-prepared rapid acquisition of gradient echoes sequence (TR = 2200 ms; TE = 3 ms; flip angle = 15°, matrix = 256 × 256 × 160, and voxel size = 1 × 1 × 1 mm).

### 2.6 Quantification of WM lesion volumes

FLAIR hyperintense WM lesions in MRI images of T1DM and MS patients were automatically segmented following the lesion growth algorithm (LGA) included in the “Lesion Segmentation Tool” (https://www.applied-statistics.de/lst.html). T1-hypointense lesions were further identified and filled with nearby WM values in MS images using the same toolbox to enhance the tissue segmentation step in the subsequent image pre-processing for GM volume quantification (Gelineau-Morel et al., 2012).

### 2.7 Quantification of global and regional GM volumes

The Computational Anatomy Toolbox (CAT-12, version 12.6, r1450) with the most recent version of Statistical Parametric Mapping (SPM12; https://www.fil.ion.ucl.ac.uk/spm/) was used to conduct voxel-based morphometry analysis. Following the standard procedures outlined in the CAT-12 manual, T1-3D images were first visually examined for artefacts before preprocessing. These procedures included i) bias-field correction; ii) segmentation into GM, WM, and cerebrospinal fluid (CSF); iii) registration to a standard template using the DARTEL algorithm; iv) normalization of GM images to the MNI template; v) modulation of the normalized data; and vi) spatial smoothing using a kernel of 8 mm FWHM.

Tissue-specific maps acquired during the segmentation process were used to calculate the brain parenchymal fraction (BPF). As a measure of global atrophy, the BPF was computed by dividing the total of the GM and WM tissue segmentation maps by the intracranial volume (the sum of the WM, GM, and CSF volumes).

### 2.8 Neuropsychological evaluations

Neuropsychological assessments were conducted using the Edinburgh Handedness Inventory (Oldfield, 1971); the Matrix Reasoning and Forward/Backwards Digit Span Subtests of the Wechsler Adult Intelligence Scale (WAIS III) as measures of intelligence quotient (IQ), short-term memory and working memory, respectively (Wechsler, 2001); the Brief Repeatable Battery of Neuropsychological Tests, validated for the Spanish population (Sepulcre et al., 2006); the Beck Depression Inventory (Wideman et al., 2013); the State-Trait Anxiety Inventory (Spielberger et al., 1983); and the Fatigue Severity Scale (Krupp et al., 1989).

### 2.9 Statistical analysis

Statistical analyses for clinical, neuropsychological, serum and OCT data and global radiological measures were performed using SPSS 24.0 (IBM, Armonk, NY) and customized SPSS syntax routines. The Shapiro‒Wilk test was used to assess the normality of distributions of all continuous variables. For parametric variables, ANOVA with the Bonferroni post hoc correction was used, and the Kruskal‒Wallis test with Dunn's post hoc test was used for nonparametric variables. ANCOVA, the parametric Student's *t*-test, the nonparametric Mann‒Whitney *U* test and the Wilcoxon rank‒sum test were performed as appropriate for continuous variables, and χ^2^ tests were used for categorical variables. When applicable, ANCOVA model designs that included age and years of education as nuisance covariates were used to compare neuropsychological scores, serum biomarkers, and OCT data between groups.

To evaluate significant between-group regional differences in GM volume, we performed a whole-brain analysis in SPM12 using an ANCOVA design including age and total intracranial volume as nuisance covariates, implementing a voxelwise threshold *p* < 0.001 in combination with a cluster size criterion determined by familywise error (FWE) correction.

Finally, to assess significant relationships between variable measures, only those variables previously showing statistical significance or a trend towards statistical significance between groups were further selected for Pearson's partial correlation analyses, adjusted for age, educational level, or intracranial volume (when appropriate). A *p* value < 0.05 was considered statistically significant.

### 2.10 Data availability

Upon reasonable request, the corresponding author will provide the anonymized raw data investigated in the present research so that the processes and results can be replicated.

## 3 Results

### 3.1 Demographic and clinical evaluation

The main clinical and demographic characteristics of the study population are presented in Table 1. The subjects included in the three experimental groups were age- and sex-matched, and the patient groups (T1DM and MS) had similar disease durations. The results of the cellular population analysis revealed a lower lymphocyte population in the MS patients than in the HCs, but no significant differences were found in the post hoc analysis (*p* = 0.056). In addition, the basophil population was significantly different between the patient groups (*p* = 0.002) but was similar to that of HCs. In terms of biochemical parameters, only glucose levels were significantly altered between the T1DM and HCs groups (*p* < 0.001) and were also altered compared with those in the MS group (*p* < 0.001). Other parameters, such as creatinine and hemoglobin levels, were significantly different between the patient groups (*p* = 0.026 and *p* = 0.007, respectively), but no difference was found between the patient groups and the HCs group. In addition, a decrease in uric acid was found in the T1DM groups, but this decrease was not significant according to post hoc analysis. The analysis of the T1DM advanced group revealed similar results to those obtained with the early T1DM group (Suppl. Table 1).

### 3.2 sNfL and sGFAP results

**Fig. 1:**
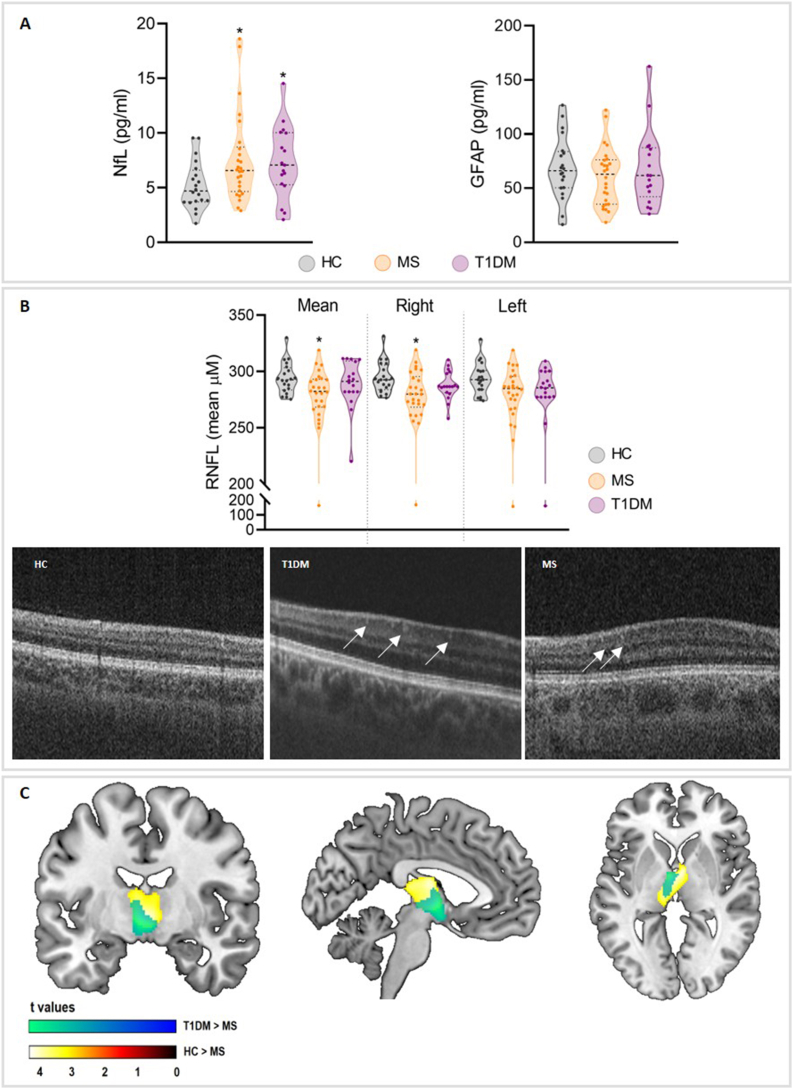
sNfL, retinal and brain alterations in T1DM and MS patients compared with HCs. **A** Representative violin plot of NfL (left chart) and GFAP (right chart) differences between study groups (both p < 0.05). **B** Violin plots showing the mean RNFL thickness in both eyes and separate representations of the right and left eyes (top chart). Representative SD-OCT images showing retinal alterations between HCs and T1DM and MS patients, such as the presence of HRS and retinal detachment in both patient groups (bottom panels). White arrows: HRS. **C** Cortical GM volume comparisons between HC and pathological groups, showing a thalamic volume reduction in MS patients compared with T1DM patients. Abbreviations: HCs, healthy control; T1DM, type 1 diabetes mellitus; MS, multiple sclerosis; HRS, hyperreflective spots; mRNFL, mean retinal nerve fiber layer; sNfL, serum neurofilament light chain; sGFAP, serum glial fibrillary acid protein; GM, grey matter.

Serum NfL and GFAP levels were measured in all the samples included in the study. The mean sNfL levels were greater in the T1DM and MS patient groups than in the HCs and showed a statistically significant trend in the group comparisons. Bonferroni post hoc analysis confirmed a significant difference in those levels between patient groups and HCs (for T1DM, *p* = 0.007; for RRMS, *p* = 0.036, see Table 1 and Fig. 1 A). Further analysis of the T1DM advanced group revealed that sNfL levels increased over time (HCs vs. T1DM advanced *p* = 0.018), highlighting a potential temporal progression in T1DM and revealing a significant trend towards significance between the MS and T1DM advanced groups (*p* = 0.065, Suppl Fig. 1 A). In the case of sGFAP, the levels were similar between all the groups analyzed in the study (Suppl. Table 1).

### 3.3 SD-OCT

The mean RNFL thickness, including the average measurement from both eyes (Table 2), demonstrated a statistically significant trend towards a slightly lower thickness across all study groups. Post hoc analysis revealed a significantly lower RNFL thickness in the MS group than in the HCs group (*p* = 0.012). Independent eye analysis revealed a specific reduction in RNFL thickness in the right eye of the MS group compared with the HCs group (*p* = 0.006). No significant differences in RNFL thickness were observed between the T1DM group and either the HCs or MS groups (Fig. 1 B).

**Table 2:** Neuropsychological and Global MRI characteristics of participants.

Variables	HC (n = 20)	T1DM (n = 18)	MS (n = 26)	p-value
**Education (years)**	17.85 (2.60)	13.00 (2.25)	12.42 (3.13)	0,000^a,b^
**Edinburgh Handedness**	17.67 (5.57)	16.64 (7.03)	18.54 (5.86)	0.640
**Manipulative IQ**				
**Matrix Design (WAIS-III)**	110.75 (12.59)	98.67 (8.34)	100.38 (13.49)	0.006^a,b^
**Fatigue Severity**				
**FSS**	25.85 (10.55)	25.94 (14.16)	45.31 (14.63)	0.000^b,c^
**Depression**				
**BDI-II**	7.90 (5.81)	10.83 (10.77)	20.08 (9.52)	0.000^b,c^
**Anxiety**				
**STAI-State**	18.55 (9.16)	16.22 (13.18)	26.85 (12.06)	0.008^c^
**STAI-Trait**	19.40 (10.92)	15.61 (10.27)	30.73 (13.73)	0.000^b,c^
**Cognition**				
**Forward Digits (WAIS-III)**	6.45(0.99)	5.94 (1.35)	5.38 (1.06)	0.073^b^
**Backward Digits (WAIS-III)**	5.10 (1.07)	4.56 (1.38)	4.56 (1.10)	0.344
**SDMT**	64.30 (12.85)	59.22 (9.83)	49.92 (10.38)	0.024^b,c^
**PASAT 3 s.**	48.90 (8.30)	38.83 (15.15)	36.92 (10.54)	0.175
**SRT Long-Term Storage**	55.55 (9.60)	52.33 (9.60)	46.27 (10.88)	0.406
**SRT Consistent Long-Term Retrieval**	49.50 (11.75)	43.83 (11.45)	39.27 (14.29)	0.934
**SRT Delayed Recall**	10.10 (1.68)	10.11 (2.35)	8.54 (2.44)	0.215
**10/36 SPART Long-Term Storage**	22.35 (4.13)	21.39 (4.34)	17.89 (5.61)	0.161
**10/36 SPART Delayed Recall**	8.05 (1.82)	7.39 (1.33)	6.15 (2.82)	0.427
**Phonetic Fluency**	12.95 (2.78)	11.11 (2.40)	10.38 (4.12)	0.459
**Semantic Fluency**	23.10 (4.59)	21.56 (6.13)	19.19 (5.06)	0.262
**MRI global measures**				
**BPF**	0.81 (0.03)	0.82 (0.02)	0.80 (0.04)	0.295
**Global Cortical Thickness (μm)**	2.49 (0.08)	2.40 (0.11)	2.41 (0.11)	0.029^a^
**Optical coherence tomography (Retinal thickness μM)**				
**Both eyes**	294.05(13.75)	283.88 (22.97)	277.59 (28.86)	0,074^b^
**Right eye**	294.45 (13.67)	284.62 (12.86)	277.42 (27.99)	0,014^b^
**Left eye**	293.66 (13.98)	273.84 (36.25)	277.74 (30.76)	0,171

*Abbreviations*: BDI = Beck Depression Inventory; BPF = brain parenchymal fraction; BRB-N = Brief Repeatable Battery of Neuropsychological Tests; FSS = Fatigue Severity Scale; GMF = grey matter fraction; HCs = healthy control; PASAT = Paced Auditory Serial Addition Test; RRMS = relapsing-remitting multiple sclerosis; SDMT = Symbol Digit Modalities Test; SPART = Spatial Recall Test; SRT = Selective Reminding Test; STAI = State-Trait Anxiety Inventory; WAIS = Wechsler Adult Intelligence Scale; WM = white matter. Values are expressed as the mean and SD. Student t tests for continuous variables were performed to assess significant differences between groups (*p* values are included). Analysis of covariance design was used to assess BRB-N score comparisons including educational level was included as a nuisance covariate. ^a^ HCs vs T1DM (*p* < 0.05), ^b^ HCs vs MS (*p* < 0.05), ^c^ T1DM vs MS (*p* < 0.05).

Nevertheless, the differences in T1DM advanced patients were significant when the eyes were analyzed separately compared with those of HCs, revealing a trend towards a reduction in RNFL thickness in T1DM advanced patients (*p* = 0.068, *p* = 0.052, right and left eye, respectively, Suppl. Table 1 and Suppl Fig. 1B and C). In brief, no RNFL differences were observed in patients with newly diagnosed T1DM, whereas the reduction was driven by the advanced T1DM subgroup, suggesting a progressive retinal involvement over the course of the disease.

SD-OCT revealed several isolated HRS localized within the middle retina, specifically in the outer plexiform layer and inner plexiform layer of the macula, with sparing of the fovea. These findings were observed exclusively in the T1DM and MS groups and not in the HC group. Additionally, SD-OCT revealed the presence of subfoveal neuroretinal detachment, characterized by a hyporreflective area beneath the neuroretina, which was associated with both the T1DM and MS groups, and being especially pronounced in the T1DM advanced group (Fig. 1 B).

### 3.4 Results from the volumetric analysis

When controlled for age and total intracranial volume, global atrophy as assessed by BPF was similar between all the experimental groups, whereas global CT was significantly different between the groups (Table 2). Post hoc analysis revealed that T1DM patients had significantly lower global cortical thinning (*p* = 0.009) and that MS patients also had a trend towards lower global cortical thinning (*p* = 0.095) than HC subjects did. Regional GM analysis (following the whole-brain approach) revealed a significant bilateral thalamic volume reduction only in the MS group compared with the T1DM (*p* = 0.001; FWE corrected) and HC (*p* = 0.001; FWE corrected) groups after controlling for age and Total Intracranial Volume (TIV, see Fig. 1 C).

### 3.5 Neuropsychological assessment

The HCs group had a greater number of years of schooling than the patient groups did (*p* < 0.001 in both groups vs. HCs). Accordingly, all analyses were adjusted for age and educational level. The neuropsychological analysis (Table 2) revealed significant differences in manipulative IQ between the patient group and HCs (T1DM *p* = 0.015, MS *p* = 0.017). Furthermore, patients with MS presented significantly greater levels of fatigue and depression than HCs did (*p* < 0.001 for both). Additionally, fatigue, depression and anxiety were significantly greater in MS patients than in T1DM patients (*p* < 0.001, *p* = 0.004, and *p* = 0.01, respectively). Cognitive assessment indicated that MS patients performed worse than HCs did (*p* = 0.02) on tasks related to short-term memory (Forward Digit Span subtest of WAIS III) and attention/information processing speed (*p* = 0.022, SDMT). However, only differences in the SDMT were observed between HCs and T1DM patients.

### 3.6 Relationships between measured variables

**Fig. 2:**
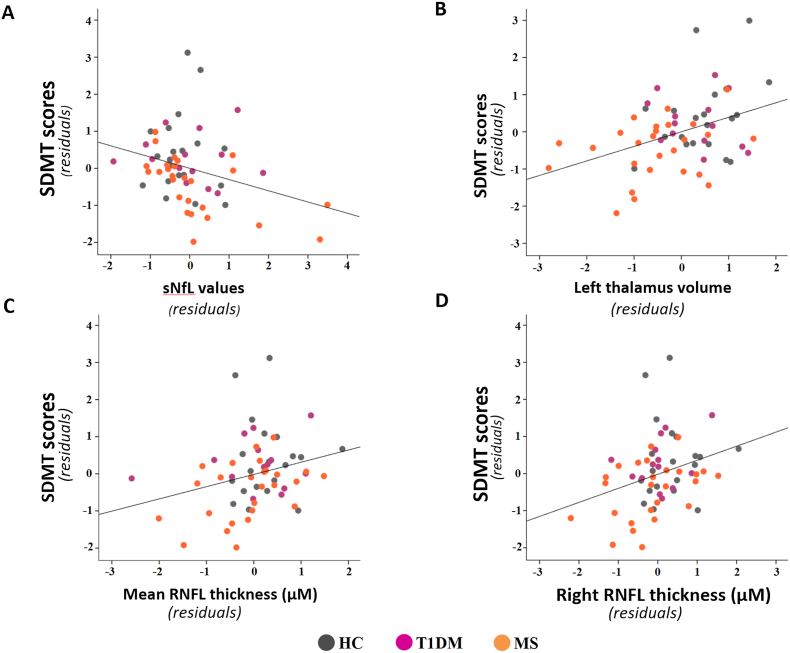
Scatterplots showing the partial correlations between variables. Partial correlation between **A** SDMT scores and sNfL levels, **B** left thalamus volume, **C** mean RNFL thickness, and **D** right RNFL thickness for all participants in the study. Partial correlations were adjusted for age, schooling, and TIV when appropriate. Both the y- and x-axes represent the standardized average residuals of age and schooling for the SDMT and sNfL, mean RNFL, and right RNFL values and of age, schooling, and TIV for the SDMT scores and left thalamus volume. Abbreviations: SDMT, Symbol Digit Modalities Test; sNfL, serum neurofilament light chain; RNFL, retinal nerve fiber layer.

Considering the entire sample, partial correlations adjusted for age, schooling, and TIV (when appropriate) revealed that higher cognitive status, as revealed by SDMT performance, was significantly associated with several parameters: lower sNfL (r = −0.272, *p* = 0.041); greater mean (r = 0.275, *p* = 0.038) and right eye (r = 0.317, *p* = 0.016) RNFL thicknesses; and left thalamus volume (r = 0.352, *p* = 0.007). An explorative analysis performed separately for each group revealed that these significant associations between the variables described remained strongly significant only in the MS group (Fig. 2).

## 4 Discussion

This study compared the clinical, cognitive, and neurological statuses of newly diagnosed T1DM and MS patients. Our findings revealed elevated sNfL levels in both diseases, supporting early neuroaxonal damage and compromised blood-brain and blood-retinal barrier integrity characteristic of autoimmune neuroinflammation (Aubé et al., 2014). Impaired cognitive processing speed was clearly associated with higher sNfL levels, retinal thinning, and reduced left thalamic volume, particularly in MS patients. Collectively, these findings underscore the value of multimodal biomarkers for early detection and monitoring of cognitive and neurological dysfunction in autoimmune diseases.

The observed reduction in lymphocyte percentages among MS patients can likely be attributed to the widespread use of DMTs, particularly high-effective DMTs, which were employed by more than half of the MS cohort in this study. These treatments are known to partially reduce circulating lymphocyte counts as part of their immunomodulatory mechanism (Oreja-Guevara et al., 2024). Notably, the predominant use of high-efficacy DMTs in our MS cohort represents a significant methodological consideration when interpreting group comparisons. These agents may have suppressed immunological indices, modulated neuroaxonal biomarker release, and influenced the magnitude of structural brain alterations, potentially underestimating disease-intrinsic neurodegeneration in untreated MS. Consequently, our findings reflect the phenotype of pharmacologically-treated MS rather than untreated disease, which should be considered when extrapolating mechanistic conclusions to broader MS populations with variable treatment exposure. While post hoc lymphocyte analyses did not reveal significant differences, the trends align with established literature reporting shifts in lymphocyte subsets, including T helper cells, in both T1DM and MS patients (Herold et al., 2024; Liu et al., 2022). This underscores the complex immunological landscape influenced by therapeutic interventions and highlights the need for further investigation into how DMTs modulate immune cell dynamics in MS.

In T1DM patients, early metabolic alterations are primarily reflected by changes in glucose levels, which are characteristic of early disease stages and tend to worsen with disease progression (Alqahtani et al., 2022). However, no significant alterations in other metabolic parameters were detected, likely due to the early disease stages and adequate metabolic control at the time of assessment. Nonetheless, evidence suggests that these parameters may become altered as T1DM progresses, likely driven by renal impairment associated with disease progression (Chutani and Pande, 2017).

A key finding was the elevated sNfL levels in both T1DM and MS patients compared with HCs. In MS, NfL is an established biomarker of axonal damage, disease progression, and response to therapy, providing robust evidence of neurodegeneration (Bittner et al., 2021). Our findings extend the relevance of sNfL to T1DM, aligning with emerging studies linking elevated NfL levels to glycemic levels (Thota et al., 2022) and peripheral complications such as diabetic neuropathy (Maalmi et al., 2022). The rise in sNfL levels observed with longer disease duration in T1DM patients underscores its potential as a biomarker for cumulative neurodegenerative burden. To characterize the temporal trajectory of neurodegeneration in T1DM, we examined an exploratory cohort with advanced disease duration. Notably, sNfL concentrations were significantly elevated in advanced T1DM patients compared to healthy controls, indicating that neuroaxonal injury accumulates progressively with prolonged disease exposure. This progression aligns with established T1DM pathophysiology, wherein chronic hyperglycemia and metabolic dysregulation cumulatively drive neuroinflammatory injury. Advanced T1DM patients also exhibited a trend toward retinal thinning, consistent with progressive microvascular and neurodegenerative complications. These exploratory findings suggest that neurodegeneration biomarkers worsen with disease progression in T1DM, supporting subsequent investigations of structural cerebral changes in longer-standing disease and their relationship to cognitive dysfunction.

Regarding GFAP, a classical marker of astroglial activation and neuroinflammation, previous studies have associated its levels with disease severity and progression, particularly during inflammatory or active phases of MS (Abdelhak et al., 2018; Barro et al., 2022; Kassubek et al., 2017). However, our study found no significant changes in serum GFAP levels in either MS or T1DM patients compared with healthy controls. This finding is possibly due to the early disease stage, where astrocytic reactivity has not yet reached a detectable threshold in peripheral blood. Furthermore, it is important to note that, our MS cohort were evaluated during clinical remission, a phase typically associated with reduced astrocytic reactivity and lower ongoing neuroinflammatory activity. Additionally, GFAP dynamics differ from those of NfL, as its release depends on chronic astrocyte activation rather than acute axonal damage, potentially delaying its peripheral elevation (Samadzadeh and Sleator, 2025). Additional factors, including inter-individual biological variability, efficient CNS clearance mechanisms and blood-brain barrier integrity, may further limit detectable serum GFAP changes. In MS, the influence of immunomodulatory treatments may additionally suppress astroglial activation while in T1DM, adequate metabolic control could modulate neuroinflammatory processes. Finally, the sensitivity limits of current serum-based GFAP assay may restrict the detection of subtle alterations at early or clinically stable stages. These results highlight the importance of disease phase and inflammatory activity when interpreting GFAP as a biomarker of neuroinflammation in MS and T1DM diseases.

Retinal imaging revealed disease-specific patterns of neurodegeneration. MS patients presented significant global thinning of the RNFL, particularly in the right eye. This was consistent with optic nerve damage from optic neuritis and broader neurodegenerative processes common in MS. NfL has been proposed as a marker of inflammation and visual outcome in different optic diseases, such as neuritis and glaucoma (Modvig et al., 2016; Woltsche et al., 2023). In contrast, T1DM patients showed no significant RNFL thinning compared with HCs; however, trends in advanced T1DM patients suggested the onset of retinal damage, likely related to chronic hyperglycemia and microvascular complications. In T1DM, the relationship between NfL levels and RNFL thickness remains unclear. These retinal changes align with diabetic retinopathy and emphasize the cumulative impact of metabolic and vascular stress on neurodegeneration. Furthermore, increased NfL levels have been associated with inner retinal neurodegeneration and outer retinal thickening (Garzone et al., 2022), matching the presence of HRS in those areas in our patient, suggesting NfL as a marker of retinal damage. Additional abnormalities, including HRS and subfoveal neuroretinal detachment, were identified in both the T1DM and MS groups, and being especially pronounced in the T1DM advanced group. These findings, indicative of localized inflammation and blood‒retinal barrier dysfunction (Vujosevic et al., 2020), suggest shared pathways of vascular compromise and neuroinflammation. While RNFL thinning was more pronounced in MS patients, the structural changes observed in advanced T1DM patients underscore the progressive nature of retinal damage across both conditions. These findings reinforce the utility of combining retinal imaging with systemic biomarkers such as NfL to monitor disease progression and neurodegeneration. While the role of neurodegeneration in MS is well studied, with established imaging markers and clinical correlations, much less is known about its presence and progression in T1DM patients.

Compared with T1DM patients, MS patients presented a bilateral loss in thalamic GM volume, confirming earlier findings indicating that thalamic atrophy is a sensitive and early neurodegenerative process in MS (Cruz-Gomez et al., 2021; Filippi and Rocca, 2010). Accumulating evidence highlights thalamic GM atrophy as a key radiological marker of cognitive impairment in MS. Numerous MRI studies have linked cognitive dysfunction with thalamic damage, typically signaling early disease progression (Amin and Ontaneda, 2021; Eijlers et al., 2018).

Although studies investigating structural regional cerebral effects in T1DM patients are scarce, some highlight that T1DM may lead to GM reduction in the thalamus, cortical regions and cerebellum. These changes may offer insight into associated cognitive deficits that could emerge throughout the course of the disease (Hansen et al., 2022; Liu et al., 2020; Moulton et al., 2015). Furthermore, these findings seem to be more evident as the disease progresses or in patients with more years of diabetes. In contrast to previous studies, T1DM patients in our sample did not show signs of generalized brain atrophy or reduced GM volume, likely because they were in the early stages of disease progression after diagnosis. However, these findings should be interpreted with caution, as T1DM patients exhibit high disease heterogeneity, with varying progression rates from the time of clinical presentation or glucose control complications even in short-term stages. Therefore, more research is needed to understand the mechanisms underlying GM atrophy and its role in the development of cognitive impairment, particularly in young and middle-aged T1DM patients with similar clinical characteristics.

There is ongoing debate on the neurological mechanism that may increase the susceptibility of the thalamus to T1DM and MS. Animal studies have shown that the anterior thalamic and hypothalamic nuclei have notably high levels of insulin receptor expression (Bondy et al., 1992), suggesting increased susceptibility of the thalamus to damage in T1DM patients. Furthermore, whilst thinning of the RNFL in MS is well-established as reflecting neuroaxonal injury with subsequent structural thalamic consequences, the relationship between retinal changes and thalamic volume in early T1DM remains preliminary. Although retrograde and transsynaptic degeneration mechanisms have been documented in MS, where axonal damage spreads along visual pathways to the thalamus, similar mechanisms in early T1DM patients merit cautious interpretation given the modest magnitude of observed changes. These observations require confirmation in longitudinal studies with larger cohorts (Mirmosayyeb et al., 2025; Özcan et al., 2023).

As mentioned, strong evidence from MS studies shows that various processes over the course of the disease, primarily related to GM lesions and brain atrophy, lead to cognitive network dysfunction and can result in clinically important cognitive impairment (Musen et al., 2006). The effects of T1DM on cognitive decline remain controversial, although extended hyperglycemia and recurrent episodes of hypoglycemia have both been implicated in T1DM-related cognitive function (Diabetes Control and Complications Trial/Epidemiology of Diabetes Interventions and Complications Study Research Group et al., 2007; Musen et al., 2006). Interestingly, the SDMT was the most sensitive cognitive test in the neuropsychological assessment and showed robust partial correlations, with left thalamic GM atrophy, RNFL thinning, and increased neuroaxonal loss measured by sNfL. Although previous evidence has emphasized the use of the SDMT as a sentinel and screening tool to detect key deficits underlying cognitive impairment in MS (Van Schependom et al., 2014, 2015), our data also indicate that information processing speed could be not only a widely affected domain in T1DM patients but also one of the first cognitive deficits to emerge in T1DM patients.

The SDMT is a visual task that demands not only information processing speed but also attention, visual perception and scanning abilities. The associations between SDMT performance, thalamic atrophy and RNFL thinning may indicate underlying pathological mechanisms affecting both visual processing and greater cognitive function. In addition, sNfL and RNFL thinning, which can be considered markers of neuroaxonal damage, may reflect pervasive early neurodegenerative processes in both T1DM and MS, underlying the manifestation of further cognitive dysfunctions in these patients. Together, our findings suggest that the associations between SDMT performance and sNfL, retinal thinning, and thalamic atrophy may be robust prognostic biomarkers for neurodegeneration and may be present early in both the T1DM and MS disease courses.

Several limitations should be considered when interpreting our findings. First, data collection across multiple sessions, although less than two months per participant, may introduce inter-session variability in cognitive and biomarker measurements; however, this inter-session interval is substantially shorter than that typically observed in comparable multi-modal neuroimaging studies, mitigating potential confounding from disease progression. Importantly, all participants remained clinically stable throughout the study period, with MS patients evaluated during remission, which mitigates potential confounding from disease progression. Second, the MS cohort was predominantly treated with high-efficacy DMTs, which attenuate lymphocyte counts and neuroinflammatory activity. This immunomodulatory effect may have suppressed certain biomarker elevations and structural changes in MS patients, potentially underestimating the magnitude of neurodegeneration in untreated disease. Third, the study sample size, whilst adequate for detecting group-level differences in our primary analyses, limits statistical power for exploratory subgroup comparisons and may particularly compromise the reliability of observed correlations between serum biomarkers and neuroimaging measures. Furthermore, the modest sample may restrict generalizability to broader MS and T1DM populations with varying clinical characteristics and disease severity, requiring confirmation in larger independent cohorts. Finally, the exploratory advanced T1DM cohort was assessed for serum biomarkers and cognitive function but lacked comprehensive neuroimaging data, precluding direct structural-functional correlation analysis in this group. Despite these constraints, our findings provide robust evidence for shared and distinct neurodegeneration mechanisms between early-stage T1DM and MS, with implications for future longitudinal and neuroimaging-focused investigations.

In summary, our findings revealed a reduction in lymphocyte percentages among MS patients, likely due to high-efficacy treatments that lower circulating lymphocyte counts. Notably, both diseases were characterized by elevated sNfL levels compared with healthy controls, reflecting early neurodegenerative changes. In T1DM, these alterations may worsen with disease progression and are accompanied by retinal thinning. Additionally, bilateral loss in thalamic GM volume was found in MS patients but not in T1DM patients, indicating that thalamic atrophy is a sensitive and robust neurodegenerative process for recently diagnosed patients with MS. Remarkably, elevated sNfL levels, retinal thinning, and loss of thalamic volume were linked to a lower SDMT performance, which indicates that information processing speed may be one of the first cognitive deficits to manifest in both T1DM and MS patients.

## 5 Conclusion

Our study suggests that shared and distinct mechanisms of neurodegeneration underlie cognitive dysfunction in newly diagnosed T1DM patients and MS patients. These findings reinforce the notion that both diseases exhibit overlapping mechanisms of immune-mediated damage and neuroinflammatory and neurodegenerative processes that impact the future development of disability and progressive neurological complications. Our findings demonstrate that combining systemic measures and blood and imaging markers can improve the accuracy of detecting cognitive impairment, highlighting the clinical utility of cross-modal biomarkers in recently diagnosed and mildly disabled patients with T1DM and MS.

## CRediT authorship contribution statement

**Fátima Cano-Cano:** Writing – review & editing, Writing – original draft, Visualization, Validation, Software, Methodology, Investigation, Formal analysis, Data curation, Conceptualization. **Álvaro J. Cruz-Gómez:** Writing – review & editing, Visualization, Supervision, Software, Resources, Project administration, Methodology, Investigation, Funding acquisition, Formal analysis, Data curation. **Lucía Forero:** Writing – review & editing, Supervision, Resources, Funding acquisition, Data curation, Conceptualization. **Almudena Lara-Barea:** Writing – review & editing, Investigation, Data curation, Conceptualization. **Elena Lozano-Soto:** Writing – review & editing, Investigation, Data curation. **Raúl Espinosa-Rosso:** Writing – review & editing, Resources, Investigation, Data curation, Conceptualization. **Raúl Rashid-López:** Writing – review & editing, Resources, Investigation, Data curation. **Eduardo Alcalde-Vílchez:** Writing – review & editing, Methodology, Investigation, Formal analysis, Data curation. **Pablo Álvarez-Ramos:** Writing – review & editing, Methodology, Investigation, Formal analysis, Data curation. **Manuel Aguilar-Diosdado:** Writing – review & editing, Validation, Supervision, Resources, Methodology, Funding acquisition, Data curation, Conceptualization. **Ana I. Arroba:** Writing – review & editing, Visualization, Validation, Supervision, Resources, Methodology, Investigation, Funding acquisition, Data curation, Conceptualization. **Javier J. González-Rosa:** Writing – review & editing, Writing – original draft, Visualization, Validation, Supervision, Software, Resources, Project administration, Methodology, Investigation, Funding acquisition, Formal analysis, Data curation, Conceptualization.

## Funding statement

This work was supported by the European Regional Development Fund and the Spanish Ministry of Science, Innovation and Universities (grants RTI2018-096951-A-I00 and PID2023-147093OB-I00), the Spanish Ministry of Economy and Competitiveness (grant: RYC-2015-18467), the Department of Health and Families of the Regional Government of Andalusia (grant: PI-0036), and the Carlos III Health Institute (grant: PI22-01718).

## Declaration of competing interest

The authors declared the following potential conflicts of interest with respect to the research, authorship, and/or publication of this article: LF received speaker fees and travel support from Biogen, Merck Serono, Novartis, Sanofi, Teva, and Roche. RE-R received speaker fees and travel support from Teva, AbbVie, Zambon, BIAL, Italfarmaco, Biogen, Merck Serono, Novartis, Sanofi, and Roche. RR-L received speaker fees from Teva, AbbVie, Lundbeck, Zambon, BIAL, Lundbeck, and Italfarmaco. JJG-R have received speaker fees from Lundbeck. The remaining authors state that no commercial or financial relationships that might be considered potential conflicts of interest existed during the research.
