# Supplementary material for: Comparative profiling of neurological and biomarker status in type 1 diabetes and multiple sclerosis: A cross-sectional observational study
Source: Brain Behav Immun Health. 2026 Mar 3;53:101210. doi: 10.1016/j.bbih.2026.101210 (PMC12993890; doi:10.1016/j.bbih.2026.101210)
Supplement: Multimedia component 1 [file mmc1.docx]

**Supplemental materials**

| **Supplementary table 1. Demographic, cellular, biochemical parameters and retinal thickness.** | | | | | |
| --- | --- | --- | --- | --- | --- |
| **Variables** | **HC (n=20)** | **T1DM (n=18)** | **MS (n=26)** | **T1DM advanced (n=12)** | **p-value** |
| **Demographic parameters** | | | | | |
| **Age** | 35,4 (6,71) | 37,00 (12,66) | 40,73 (10,34) | 35,83 (9,72) | 0,223 |
| **Sex** |  |  |  |  |  |
| **Male/female** | 10/10 | 10/8 | 9/17 | 6/6 | - |
| **Female %** | 50 | 44,45 | 65,38 | 50 | - |
| **Disease duration (years)** | - | 3,21 (1,5) | 4,31 (2,91) | 21,83 (11,35) | 0,00 ^e, f^ |
| **Clinical parameters** | | | | | |
| **EDSS** | - | - | 1,48 (1,36) | - | - |
| **Hg1B** | - | 6,9 (0,01) | - | 8,1 (0,01) | - |
| **Cellular populations** |  |  |  |  |  |
| **Leukocytes** | 5,8 (1,19) | 6,28 (1,48) | 6,15 (2,76) | 8,64 (2,79) | 0,074 |
| **Erythrocytes** | 4,53 (0,31) | 4,84 (0,45) | 4,64 (0,44) | 4,77 (0,5) | 0,140 |
| **Eosinophils %** | 3,06 (2,61) | 4,6 (3,17) | 2,88 (1,98) | 4,79 (1,7) | 0,077 |
| **Basophil %** | 0,56 (0,27) | 0,79 (0,27) | 0,41 (0,31) | 0,8 (0,33) | 0,001  ^c^ |
| **Monocytes %** | 7,9 (1,52) | 7,67 (1,83) | 9,55 (2,93) | 7,01 (1,54) | 0,05 |
| **Neutrophils %** | 52,45 (7,89) | 53,68 (11,67) | 60,93 (15) | 53,34 (6.10) | 0,140 |
| **Lymphocytes %** | 35,78 (7,24) | 33,22 (9,89) | 26,23 (13,43) | 34,06 (4,93) | 0,032 |
| **Platelets** | 245,82 (33,48) | 223,73 (37,16) | 235,25 (66,94) | 289,43 (65,21) | 0,120 |
| **Biochemical parameters** |  |  |  |  |  |
| **Glucose** | 90,91 (12,98) | 178,94 (57,37) | 89,79 (15,09) | 195,86 (140,08) | 0,00 ^a, c, d, e^ |
| **Cholesterol** | 190,55 (33,88) | 178,06 (47,81) | 174,73 (30,95) | 154,14 (67,63) | 0,323 |
| **Triglycerides** | 71,4 (18,15) | 80,53 (39,84) | 94,03 (46,91) | 101,71 (45,64) | 0,450 |
| **Haemoglobin** | 13,35 (1,13) | 10,18 (7,17) | 13,9 (1,31) | 9,2 (7,38) | 0,007 ^c, e^ |
| **Uric Acid** | 5,61 (2,96) | 3,96 (0,73) | 4,97 (4,10) | 3,77 (0,67) | 0,024 |
| **Urea** | 29,56 (6,5) | 28,15 (10,74) | 31,3 (9) | 33,71 (8,62) | 0,820 |
| **Creatinine** | 0,72 (0,13) | 1,12 (0,79) | 0,75 (0,18) | 0,83 (0,1) | 0,026  ^c^ |
| **Serological measures (SIMOA)** | | | | | |
| **NfL (pg/ml)** | 5.21 (2.18) | 7.34 (3.28) | 7.59 (4.06) | 12,22 (12,5) | 0,012 ^a, b, d, e^ |
| **GFAP (pg/ml)** | 68.90 (28.72) | 68.46 (35.52) | 60.71 (26.82) | 53,97 (19,7) | 0,386 |
| **Optical coherence tomography (Retinal thickness µM)** | | | | | |
| **Both eyes** | 294.05(13.75) | 283.88 (22.97) | 277.59 (28.86) | 284,1 (16,49) | 0.132 |
| **Right eye** | 294.45 (13.67) | 284.62 (12.86) | 277.42 (27.99) | 284,02 (16,49) | 0.037 ^b^ |
| **Left eye** | 293.66 (13.98) | 273.84 (36.25) | 277.74 (30.76) | 283,18 (13,78) | 0.259 |

Abbreviations: NfL = Neurofilament light chain; GFAP = Glial Fibrilar Acid Protein; SIMOA = Single Molecular Assay. Values are expressed as the mean and SD. ANOVA or Kruskal-Wallis test were performed to assess significant differences between groups (p values are included), and Bonferroni or Duns post-hoc analysis were used for pair comparisons. ^a^ HC vs T1DM (p < 0.05), ^b^ HC vs MS (p < 0.05), ^c^ T1DM vs MS (p < 0.05), ^d^ HC vs T1DM advanced (p < 0.05), ^e^ T1DM advanced vs MS (p < 0.05), ^f^ T1DM advanced vs T1DM (p < 0.05).

**Supplementary Figure 1**

**C**


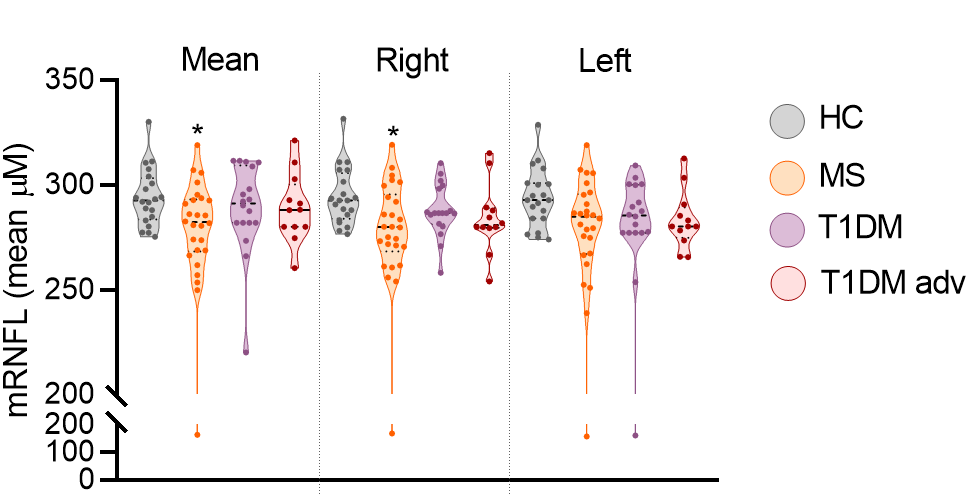

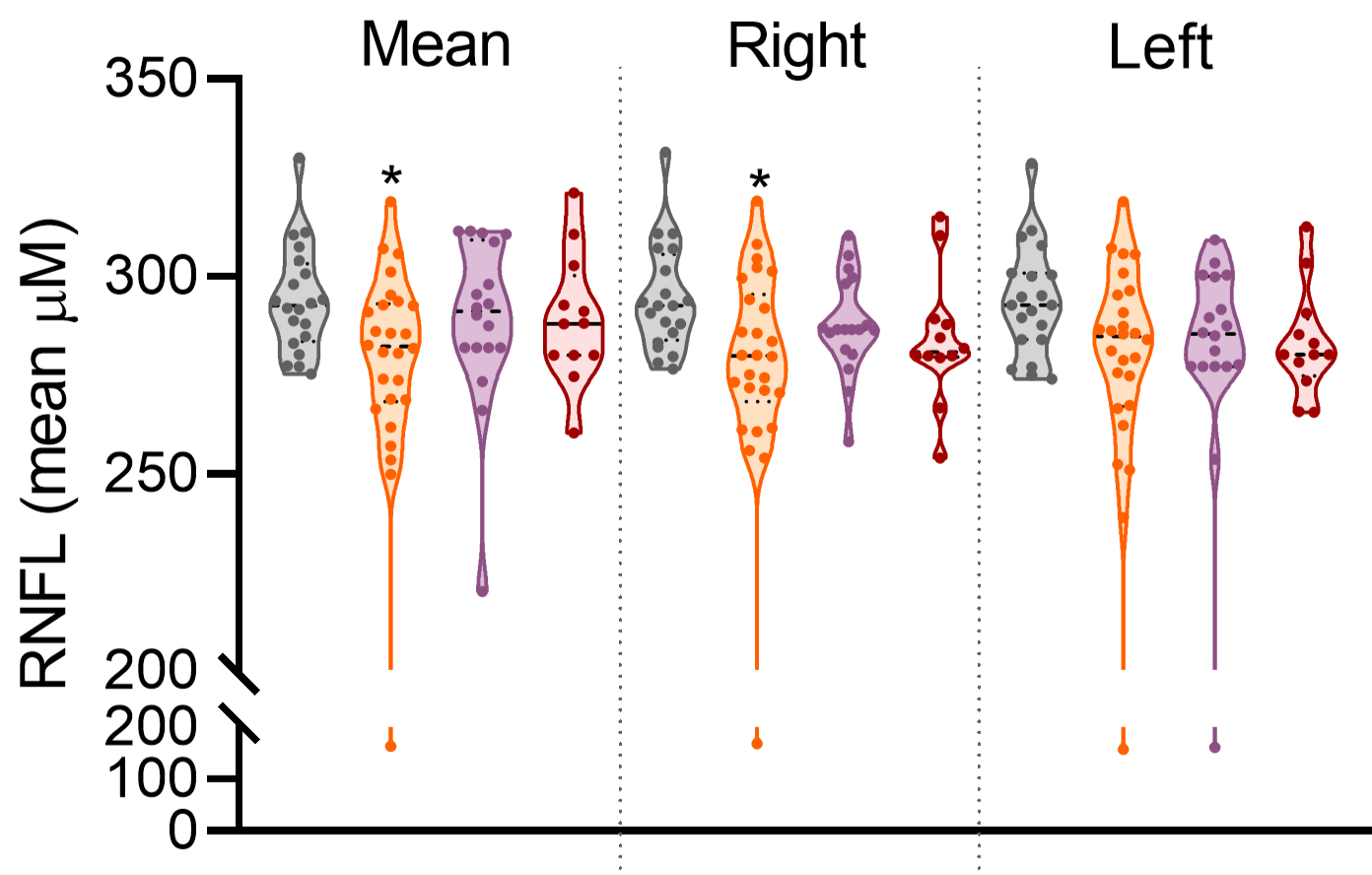


**B**


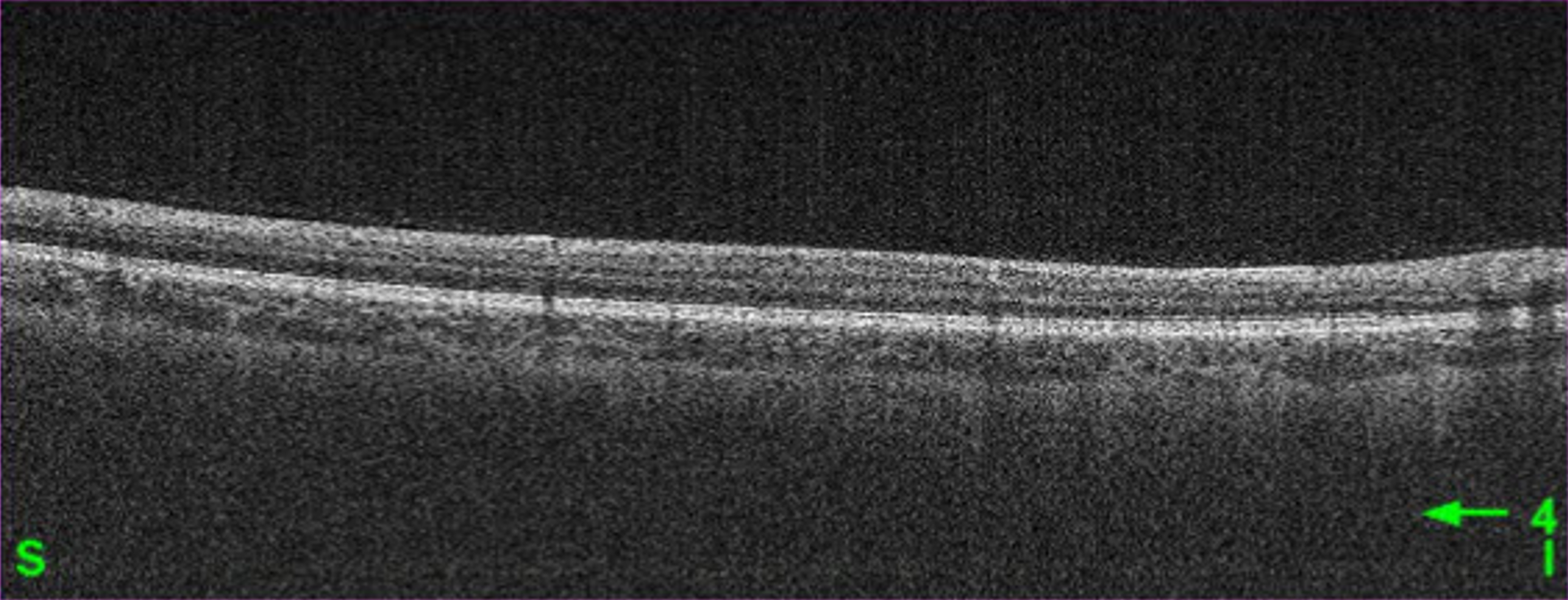


**HC**


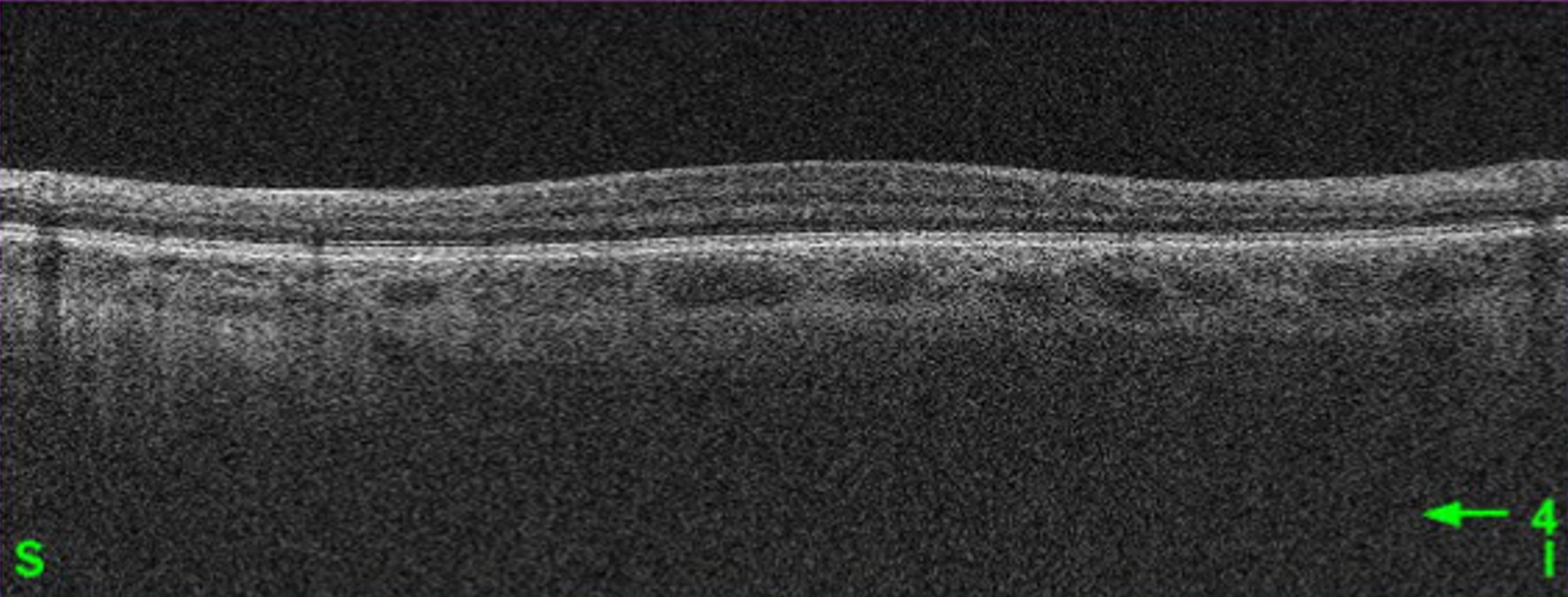


**MS**


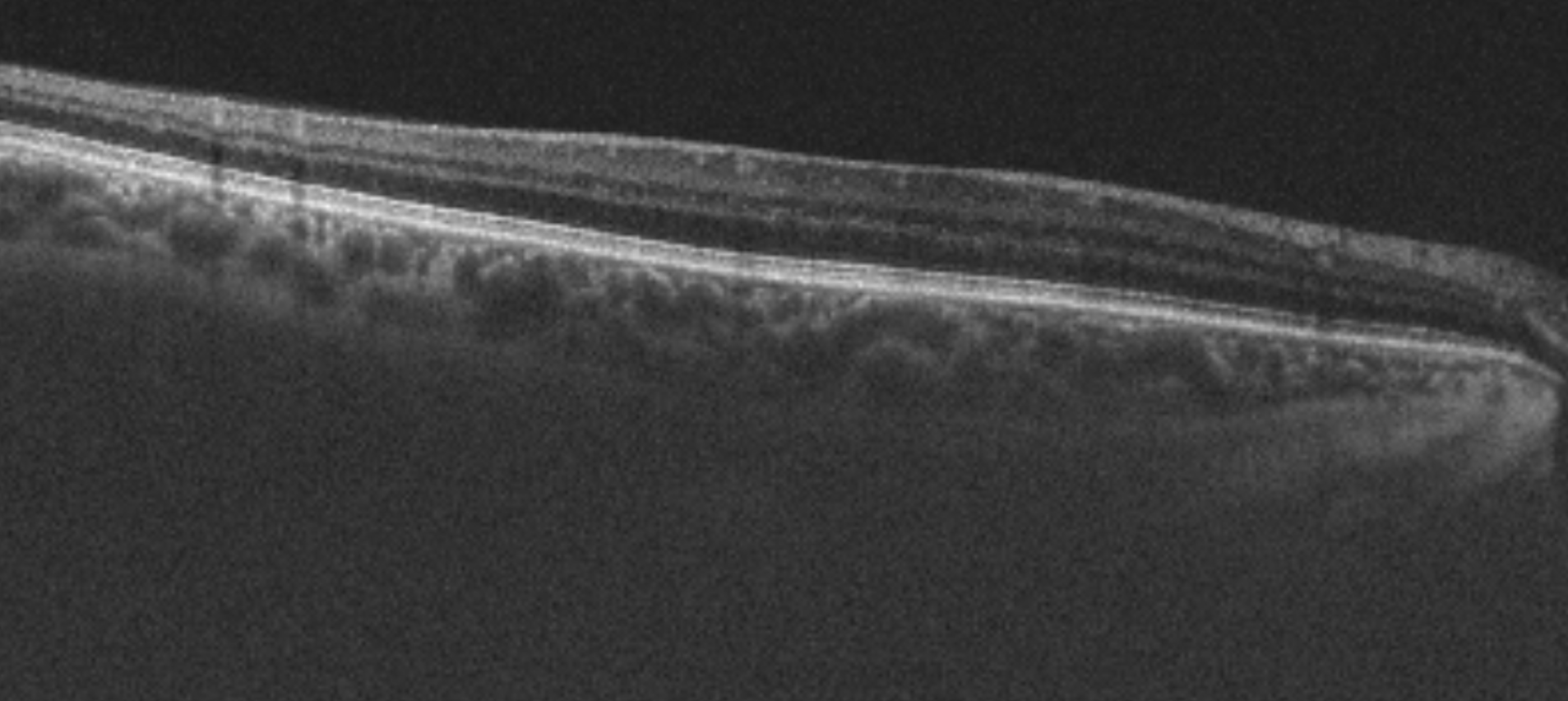


**T1DM**


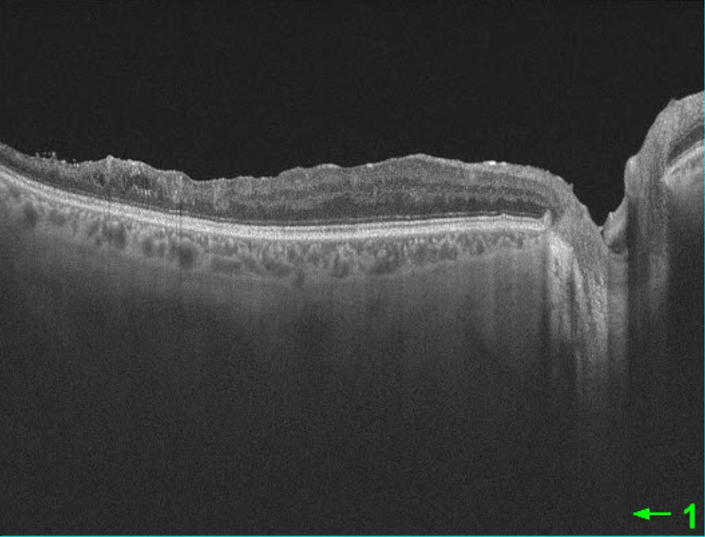


**T1DM advanced**

**A**


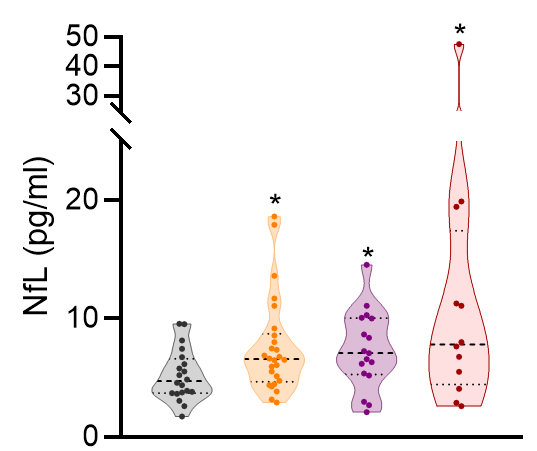

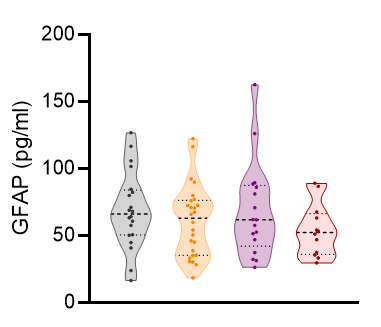

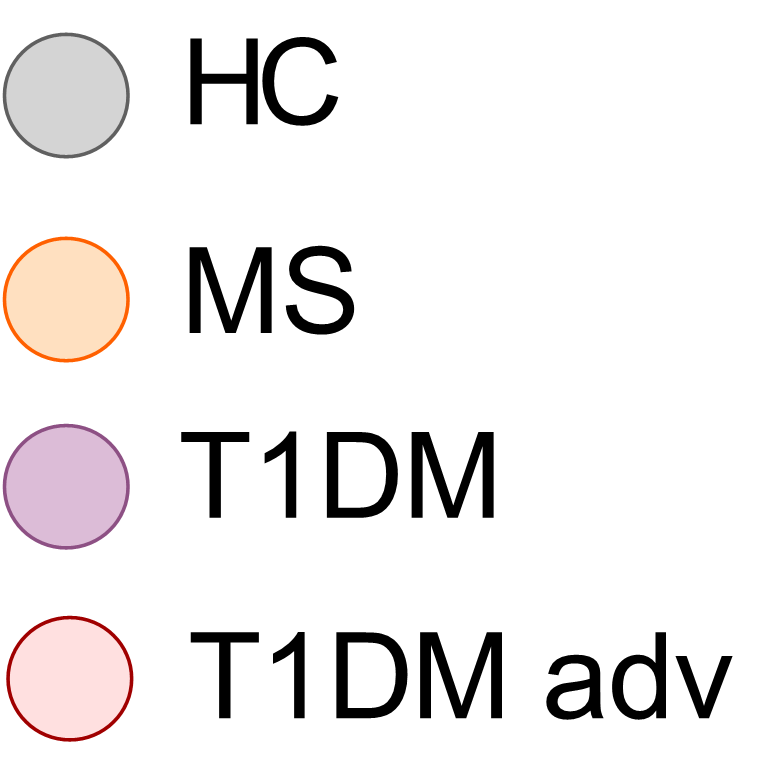

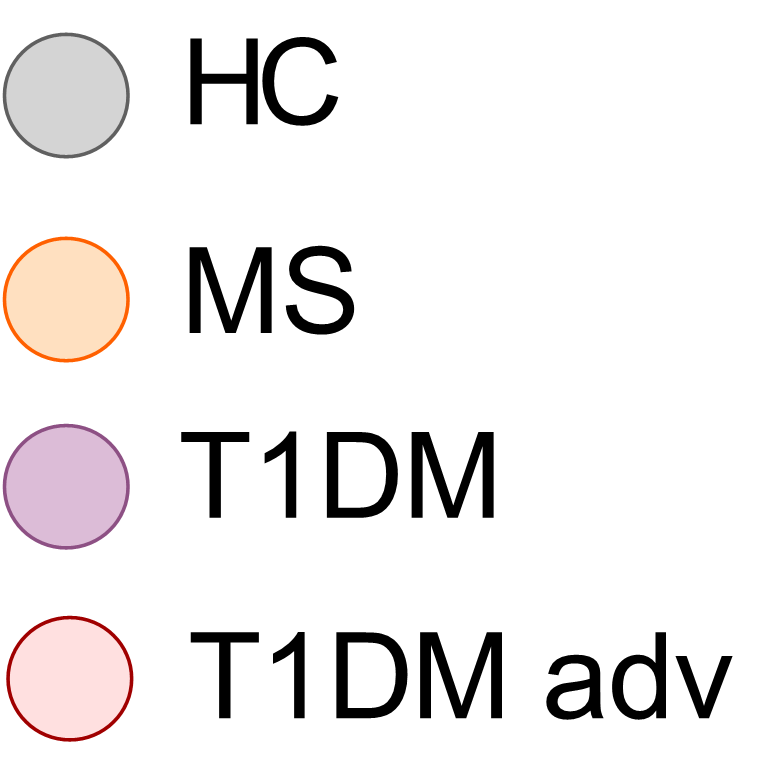

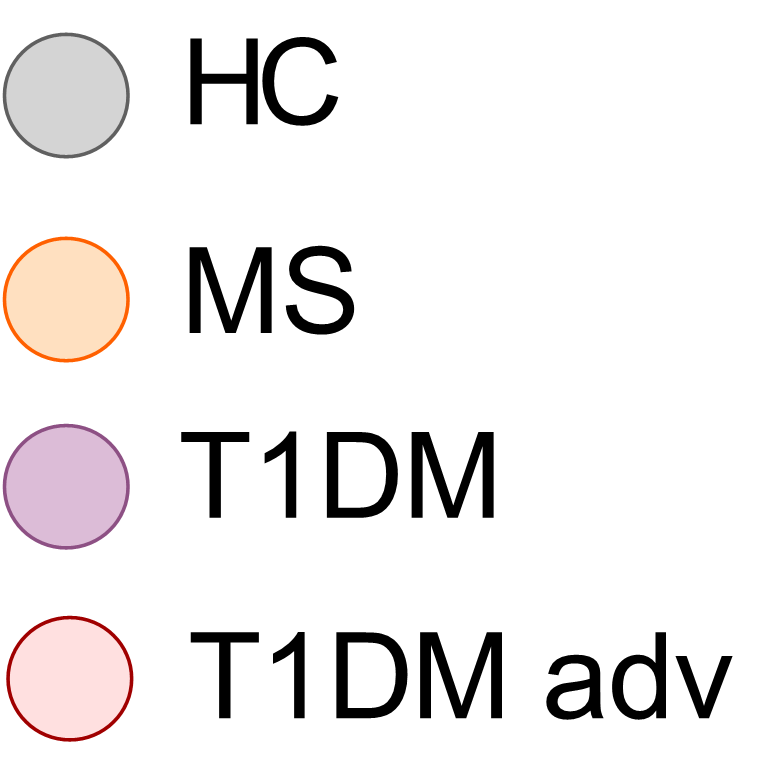

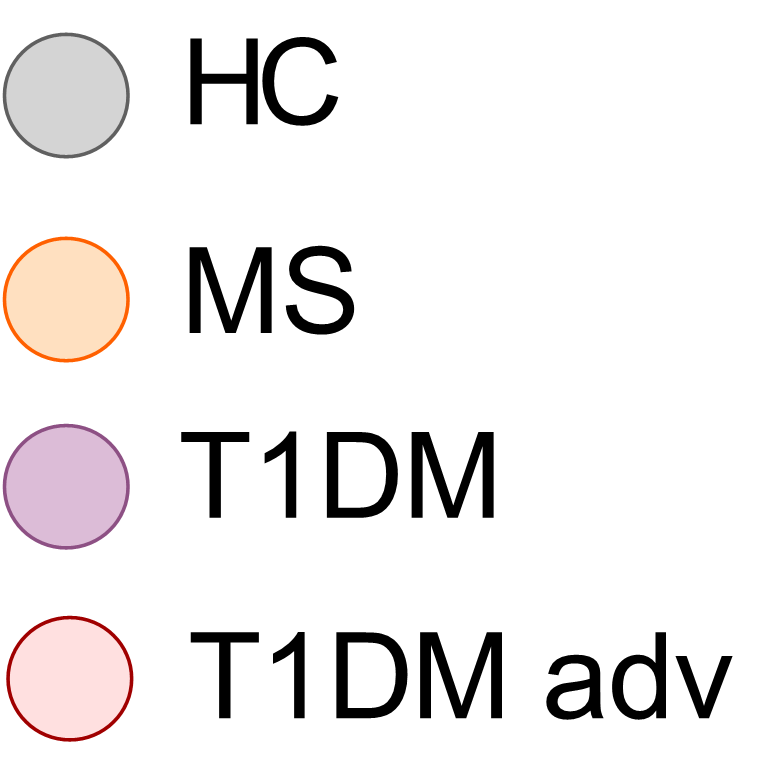


**Supplementary. Fig. 1. Retinal and sNfL, alterations in T1DM, MS and T1DM advanced patients compared with HCs. A** Representative violin plot of NfL and GFAP differences between study groups, including the advanced T1DM group (*p* < 0.05). **B** Violin plots showing the mean RNFL thickness in both eyes and separate representations of the right and left eyes. **C** Representative SD-OCT images showing retinal alterations between HCs, T1DM and MS patients, and T1DM patients in the advanced stage. There was HRS, retinal detachment and loss of retinal structure in the advanced T1DM group. White arrows: HRS. *Abbreviations*: HCs, healthy control; T1DM, type 1 diabetes mellitus; MS, multiple sclerosis; T1DM advanced, type 1 diabetes mellitus advanced; HRS, hyperreflective spots; mRNFL, mean retinal nerve fiber layer; sNfL, serum neurofilament light chain; sGFAP, serum glial fibrillary acid protein.
